# Determination of Magnetic Parameters of Maghemite (γ-Fe_2_O_3_) Core-Shell Nanoparticles from Nonlinear Magnetic Susceptibility Measurements

**DOI:** 10.1186/s11671-017-2053-5

**Published:** 2017-04-17

**Authors:** Ihor I. Syvorotka, Lyubomyr P. Pavlyk, Sergii B. Ubizskii, Oleg A. Buryy, Hrygoriy V. Savytskyy, Nataliya Y. Mitina, Oleksandr S. Zaichenko

**Affiliations:** 1 Scientific Research Company “Carat”, 202, Stryiska Str, Lviv, Ukraine; 2 Lviv Polytechnic National University, 12, S. Bandera Str, Lviv, Ukraine; 3 Ya. S. Pidstryhach Institute for Applied Problems of Mechanics and Mathematics of the NAS of Ukraine, 3b, Naukova Str, Lviv, Ukraine

**Keywords:** Superparamagnetic nanoparticles, Nanoparticles of “core-shell” type, Magnetic susceptibility, Inductive response, Non-harmonic response, 75.30.Cr, 75.47.Lx, 75.50.Dd

## Abstract

Method of determining of magnetic moment and size from measurements of dependence of the nonlinear magnetic susceptibility upon magnetic field is proposed, substantiated and tested for superparamagnetic nanoparticles (SPNP) of the “magnetic core-polymer shell” type which are widely used in biomedical technologies. The model of the induction response of the SPNP ensemble on the combined action of the magnetic harmonic excitation field and permanent bias field is built, and the analysis of possible ways to determine the magnetic moment and size of the nanoparticles as well as the parameters of the distribution of these variables is performed. Experimental verification of the proposed method was implemented on samples of SPNP with maghemite core in dry form as well as in colloidal systems. The results have been compared with the data obtained by other methods. Advantages of the proposed method are analyzed and discussed, particularly in terms of its suitability for routine express testing of SPNP for biomedical technology.

## Background

There are different applications of biocompatible magnetic nanoparticles (MNP) in biomedical technologies. The MNP can be applied to cell separation, immunoassay, magnetic resonance imaging (MRI), drug and gene delivery, minimally invasive surgery, radionuclide therapy, hyperthermia and artificial muscle applications (see [1] for example). Most of these applications require superparamagnetic state of MNP. However, nanoparticles agglomerate very easily. By this reason, the production methods of superparamagnetic nanoparticles (SPNP) are being developed. Those nanoparticles should be weakly interactive and as a result incapable to stick together.

One of the different ways to avoiding MNP agglomeration is the production of composite particles of the core-shell type [2]. The particle core made of iron oxide is superparamagnetic, and the polymeric shell does not allow them to agglomerate. The polymeric shell serves also to functionalize nanoparticles for specific applications [1, 2]. The diameter of the nanoparticles has a high impact on the imaging quality in magnetic particle imaging. Thereby, only the magnetic core of the particle contributes to the measured signal. Thus, only the diameter of the magnetic core is important for magnetic particle imaging, but not the total size of particles. Besides, most common techniques measure the total size of the particles. It is important to have instruments for measurement of nanoparticle parameters such as magnetic moment, size and size distribution for their practical use. Complex characterization of MNP usually requires package of measurements, such as scanning- or transmitted electron microscopy (SEM, TEM), vibrating sample method (VSM) and X-ray diffraction (XRD), which is difficult to use in routine investigations. That is why simple and easy express methods are needed in investigation of magnetic nanoparticle properties.

To date, several magnetic detection techniques have been employed to measure the magnetic response of the particles with respect to a magnetic excitation field. One of them is susceptometry, i.e., detection of the response to a magnetic excitation at the fundamental frequency, the technique allowing to determine quantitatively the magnetic particle concentration in a test volume [3, 4]. The other is the relaxometry which is based on recording the time transient of the magnetic response of the particles during the off-time of a pulsed excitation field. The technique allows making a distinction between bound and unbound magnetic particles [5]. Another technique is based on frequency mixing at the nonlinear magnetization curve of superparamagnets. This detection technique for MNP is used in immunoassay and magnetic particles determination in liquids [6–9].

Our approach for SPNP characterization is based on a nonlinear susceptibility measurement [10]. The technique is similar to that of susceptometry, but measurements are made not on fundamental frequency but on the second harmonic of the excitation signal. It was first investigated in [11]. As it was shown, the method allows evaluating of magnetic moment and concentration of particles very easily but it was based not only on the assumption that all the particles are superparamagnetic but also they all were equal in size. The second assumption cannot be realistic since no existing method is capable of producing the monodisperse nanoparticles. In the given work, we analyze the expansion of this approach for determination of the nanoparticle size distribution parameters in the frame of lognormal distribution model.

## Method

**Fig. 1:**
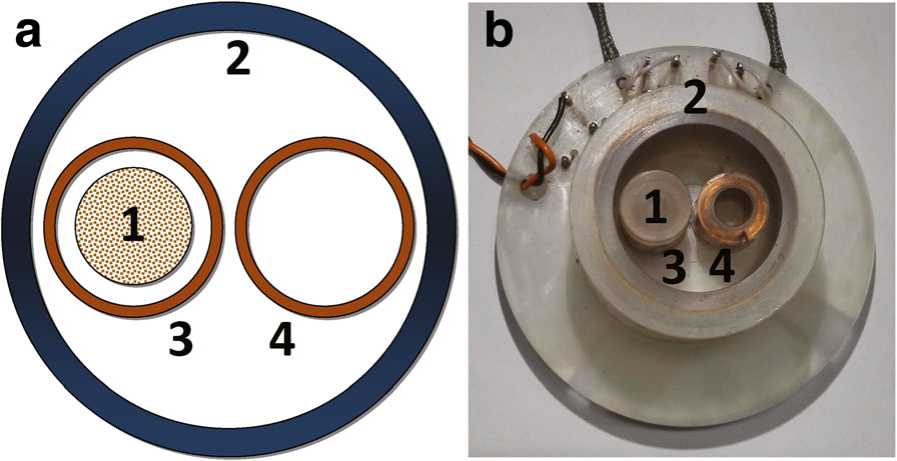
Schematic mutual position of coils (**a**) and measuring cell (**b**). *1*—sample, *2—*excitation coil, *3—*signal coil, *4*—compensation coil

Method of nonlinear magnetic susceptibility measurement is based on the simultaneous action of two magnetic fields (quasi constant–bias field and AC magnetic field–excitation field) on the ensemble of magnetic nanoparticles. The amplitude of the excitation field is supposed to be much smaller than the value of the bias field. The response of SPNP ensemble is detected by electromagnetic induction method. Schematically detecting unit is shown on Fig. 1. Sample 1—the ampoule with SPNP, is placed into the AC excitation field provided by excitation coil 2. Detection of the inductive response is implemented by sensing coil 3 covering by winding the sample and with axis parallel to the axis of the excitation coil 2. Electromotion force (EMF) induced in the sensing coil 3 is provided both by change of the excitation magnetic field and by change of the ensemble of SPNP magnetization component along with the sensing coil 3 axis. Compensation coil 4 without sample, identical to the sensing and with the axis parallel to the axis of the sensing coil, is connected in opposite to the sensing coil helping to exclude the part of EMF induced by the change of the excitation field from the measured signal.

Taking into account that the sample is under the action of the constant field *H* and the harmonic excitation field of low amplitude *h*(*t*) = *h*_0_sin(ω*t* + Δφ) and directions of the fields coincide, the measured induced EMF signal can be presented as:

1 $$ \begin{array}{c} U(t)= nV\left(\upchi (H)+\frac{\partial \upchi (H)}{\partial H}{h}_0 \sin \left(\upomega t+\varDelta \upvarphi \right)\right)\upomega {h}_0 \cos \left(\upomega t+\varDelta \upvarphi \right)=\\ {}= nV\upchi (H)\upomega {h}_0 \cos \left(\upomega t+\varDelta \upvarphi \right)+\frac{nV}{2}\frac{\partial \upchi (H)}{\partial H}\upomega {h}_0^2 \sin 2\left(\upomega t+\varDelta \upvarphi \right).\end{array} $$

where *n* is the winding density of the sensing (compensation) coil, *M* sample magnetization component along the sensing coil axis, *V* the volume of the sample and *χ*(*H*) magnetic susceptibility of the sample.

As one can see, the resulting response signal is composed not only from the fundamental but also from the second harmonic of the excitation being the result of the nonlinearity of the magnetic medium of the core.

If the sample is composed of nanoparticles of equal dimensions, they as a result have equal magnetic moments and their magnetization is well described by the Langevin theory [12]:

2a $$ M(H)= N m(H)= N{\left.{\upmu}_p L(x)\right|}_{{}_{x=\frac{\upmu_p H}{kT}}} $$

2b $$ L(x)=\frac{e^x+{e}^{- x}}{e^x-{e}^{- x}}-\frac{1}{x} $$

where μ_*p*_ is magnetic moment of a single particle at a temperature *T*, *N* concentration of magnetic particles in a sample, *x* = μ_*p*_ *H*/(*kT*), *k* the Boltzmann constant and *L*(*x*) the Langevin function. In assumption that nanoparticles are spherical, their magnetic moment μ_*p*_ and dimensions at a given temperature can be found from the fit of the expression (2) to experimental magnetization curve of the ensemble of SPNP [10, 13]. This can be done as well by fitting the field dependencies of the harmonics of the induction response signal (1). In assumption that nanoparticles are superparamagnetic and of equal dimensions, the amplitude of the first harmonic of the induction response signal (1) will be:

3 $$ {U}_{(1)}(H)= nV\upomega N\cdot \frac{\upmu_p{h}_0}{kT}{\left.\frac{dL(x)}{dx}\right|}_{x=\frac{\upmu_p H}{kT}} $$

and the amplitude of the second harmonic, being the result of nonlinearity of magnetic nanoparticles, will look like:

4 $$ {U}_{(2)}(H)= nV\upomega N\cdot \frac{1}{2}{\left(\frac{\upmu_p{h}_0}{kT}\right)}^2{\left.\frac{d^2 L(x)}{d{ x}^2}\right|}_{x=\frac{\upmu_p H}{kT}} $$

Expression (4) allows to determine magnetic moment μ_*p*_ of SPNP from the dependency *U*_(2)_(*H*) at given *n*, *V*, ω, *h*_0_ and *T*.

After the estimation of the moment of a single particle or average moment of the ensemble of the particles and in assumption that the particles are spherical, the diameter of the particle can be found from the known magnetic moment [13]:

5 $$ {d}_p={\left(\frac{6}{\pi}\frac{\mu_p}{\mu_{uc}}{V}_{uc}\right)}^{1/3} $$

where μ_*uc*_ is the magnetic moment of a unit cell and *V*_*uc*_ the volume of the unit cell.

It is evident that in real ensemble of magnetic nanoparticles, independently on the production method, the particles are not equal in size but have some distribution. Normally, the model of lognormal distribution of nanoparticles gives a good approximation to the real distribution by size. Then due to the properties of logarithmic function, both the surface and the volume of spherical particles as well as magnetic moment will have lognormal distribution:

6 $$ f\left(\mu \right)=\frac{1}{\mu \sigma \sqrt{2\pi}} \exp \left(-\frac{{\left( \ln \left(\mu /{\mu}_m\right)\right)}^2}{2{\sigma}^2}\right) $$

where μ_*m*_ and σ are the lognormal distribution parameters representing correspondingly the median value of SPNP magnetic moment distribution and the standard deviation of the magnetic moment logarithm from the logarithm of magnetic moment median value, respectively. Although in general case the saturation magnetization of magnetic nanoparticles depends on the size of the particles in a small range of the particles diameter, it can be considered equal for all the particles in ensemble.

Based on the theory of nonlinear inductive response of the SPNP ensemble described earlier, the amplitude of the second harmonic can be represented as:

7 $$ {U}_{(2)}(H)= n V\omega \frac{N}{(kT)^2}{\displaystyle \underset{0}{\overset{\infty }{\int }}{\mu}^3\frac{d^2 L(x)}{d{ x}^2} f\left(\mu \right)} d\mu $$

where $\frac{d^2 L(x)}{d{ x}^2}= \coth (x)\left({ \coth}^2(x)-1\right)-\frac{2}{x^3}$. If (7) is used to fit experimental dependence of *U*_(2)_(*H*) instead of (4), then parameters of lognormal distribution of SPNP μ_*m*_ and σ by magnetic moment can be found. Magnetic moment mean value μ_*p*_ and standard deviation *s*_*m*_ can be then obtained as:

8a $$ {\mu}_p= \exp \left({\mu}_m+{\sigma}^2/2\right) $$

8b $$ {s}_m={\mu}_p\sqrt{ \exp \left({\sigma}^2\right)-1} $$

Corresponding parameters of particle distribution by diameter in assumption that particles are spherical are related to the parameters of magnetic moment distribution by expressions:

9a $$ {d}_m={\left(\frac{6}{\pi}\frac{\mu_m}{\mu_{uc}}{V}_{uc}\right)}^{1/3} $$

9b $$ {\sigma}_d=\sigma /3 $$

Mean value of diameter *d*_*p*_ and the standard deviation *s*_*d*_ of the SPNP from the mean value can then be found from the parameters of particle distribution by diameter.

To investigate possibility of experimental determination of magnetic moment and dimensions of SPNP, the core-shell nanoparticles with core made of maghemite (γ-Fe_2_O_3_) and a polymeric shell were used. The particles were produced by homogeneous nucleation method with oligoperoxide modificator. Polymerization of monomer mixture of *N*-vinyl pyrrolidone (NVP) and glycidyl methacrylate (GMA) was performed initiated from the surface of the maghemite by means of peroxide fragments of polymeric modificator to obtain the polymeric shell on the surface of the MNP. The process is described in more details in [14, 15]. Three types of samples, obtained in two different ways, were investigated: one of the samples—M-12 in the form of dry powder, the second—M-13 in the form of water colloidal suspension after synthesis and the third one—M-13 after drying the suspension. The masses of the samples were 10.6, 4.1 and 61.1 mg. The part of dry substance in the colloidal suspension was 3% of the total mass of the sample.

**Fig. 2:**
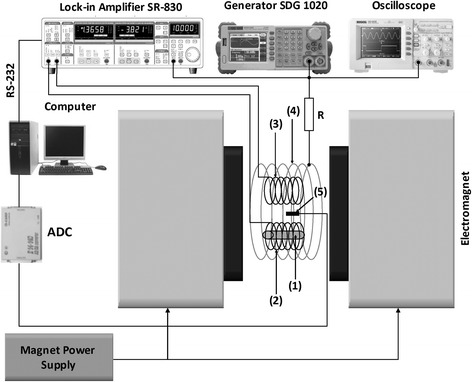
Setup for measure AC susceptibility. *1*—superparamagnetic nanoparticles, *2*—pick-up coil, *3*—compensation coil, *4*—excitation coil, *5*—Hall sensor

Schematically, the experimental setup is shown in Fig. 2. The sample 1 consisting of SPNP is located in the sensing coil 2. The sensing and identical compensation coils are placed into the excitation coil 4. The alternating magnetic field is generated by single excitation primary coil connected to the sine source via the resistor R. The two secondary coils are connected directly to the differential input of the lock-in amplifier SR830. The sample is located in the pick-up coil. The lock-in amplifier is synchronized with the field in the excitation coil making possible the measurement of the in-phase and out-of-phase components of magnetic susceptibility. Coils are placed into electromagnet which is fed by a controlled current source enabling possibility of performing constant change of the bias field with controlled rate. The field is controlled by the Hall sensor 5. By means of such setup, it is possible to measure both the fundamental and the higher harmonics of the response signal. Measurements were performed at a room temperature at amplitude of the applied field equal to 0.5 mT and frequency 11 kHz. The bias field changed in the range ±0.2 T and with rate of 0.2 mT/s.

Average magnetic moment of the ensemble of SPNP was defined by both fitting the experimental data using (4) and (7). Parameters of distribution of nanoparticle moment and consequently diameter were determined from the fit of experimental data by (7) by Levenberg-Marquardt method in assumption that nanoparticles are spherical and have equal saturation magnetization.

## Results and Discussion

**Fig. 3:**
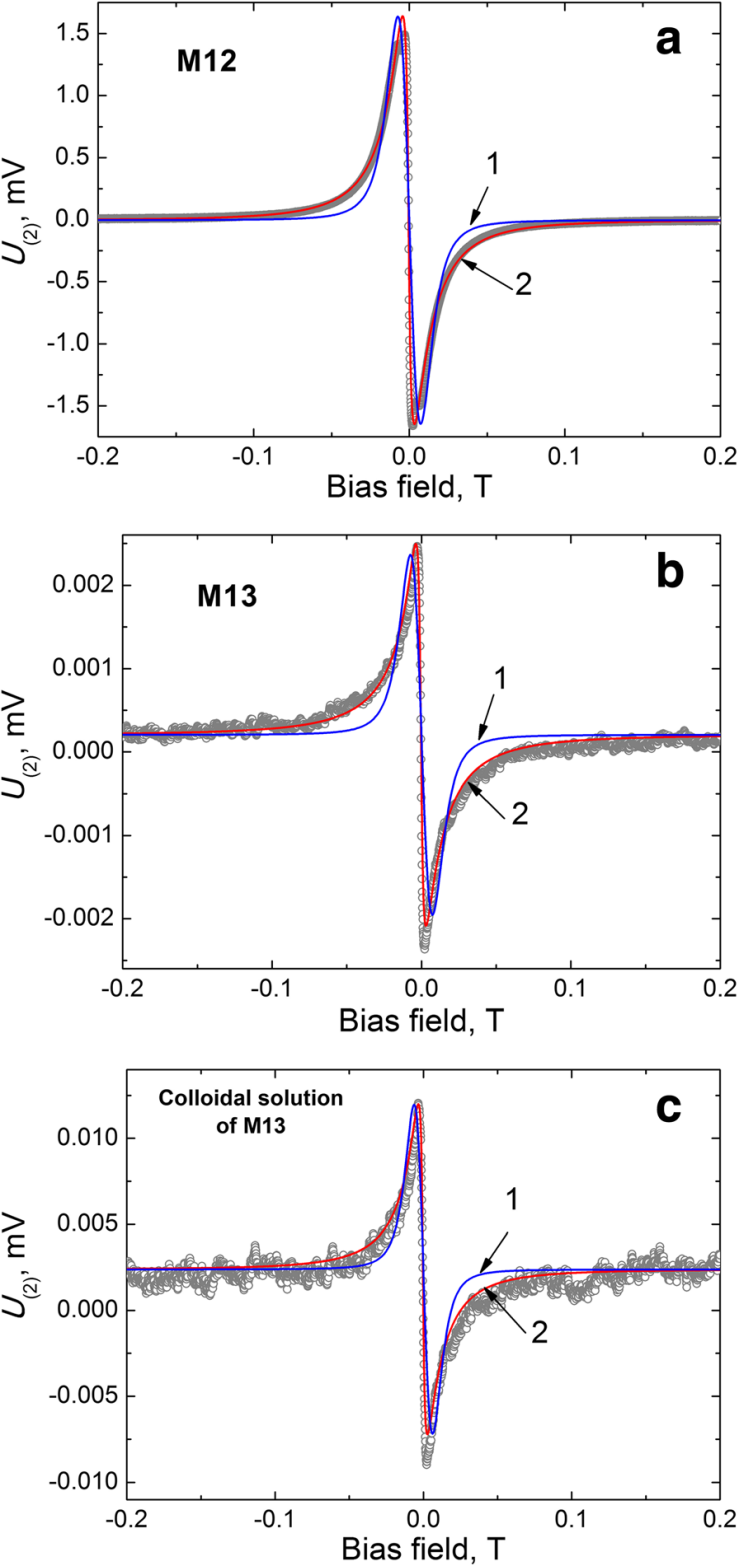
Magnetic field dependencies of the response signal 2nd harmonic (*open dots*) of the sample M12 (**a**), M13 (**b**) and colloidal solution of M13 (**c**) and fit by (4) (*blue curve 1*) and (7) (*red curve 2*)

Measured field dependence of the second harmonic response amplitude from samples of SPNP M12, M13 and colloidal solution of M13 together with fit of theexperimental data by (4) and (7) are shown in Fig. 3. As one can see from the comparison of results in Fig. 3a–c, decrease of the mass of particles causes the decrease of precision of measurements (or increasing of the noise level in the measured signal).

Fit of the experimental data by (4) in assumption that particles are equal in size gives significant disagreement between theoretical and experimental values. Approximation of the experimental data by (7) considering magnetic moment lognormal distribution gives much more precise results. Average values of magnetic moment and diameter of the particle core were determined from the distribution parameters. Obtained results are shown in Table 1 together with results of measurements by other methods: XRD, TEM, as well as with results of approximation of sample magnetization dependence measured by VSM in the field range 0.8 T [14]. Average values in the last row are shown together with deviation. The average values and deviations were found by (8). Characteristic feature of the nonlinear magnetic susceptibility dependency on the applied field as we can see is that all changes are concentrated in the narrow field range. In particular, saturation of the dependency is observed already at a field 0.2 T while corresponding changes in magnetization curve require much wider field range.

**Table 1:** Results of determination of average magnetic moment and diameter of investigated nanoparticles M12, M13 and colloidal solution of M13 by different methods

Sample	M12		M13		Colloidal solution of M13	
Determination method	μ_*p*_, [μ_*B*_]	*d*_*p*_, [nm]	μ_*p*_, [μ_*B*_]	*d*_*p*_, [nm]	μ_*p*_, [μ_*B*_]	*d*_*p*_, [nm]
Widening of X-ray diffraction peaks (Sherrer method) [14]	–	12.1 ± 0.6	–	4.8 ± 0.4	–	–
Transmission electron microscopy [14]	–	10.0 ± 2.2	–	–	–	–
Magnetization curve approximation in a field range 0.8 T [14]	18050	9.4 ± 3.9	–	–	–	–
Approximation by *U*_(2)_(*H*) in a field range 0.2 T considering lognormal distribution of particles moment and diameter	19180	8.5 ± 2.5	9 460	6.5 ± 2.4	14460	7.6 ± 2.5

As we can see, the method of determination of SPNP magnetic moment and diameter proposed in the present work gives results that conform well with results obtained by other known methods. On the other hand, the method has some advantages. The main is the possibility to determine both the magnetic properties as well as dimensions of SPNP simultaneously together with parameters of magnetic nanoparticles distribution by size. The method is applicable to the sample in shape of dry powder as well as colloidal solutions of SPNP. This method does not require independent methods of particle size determination, but it simplifies routine measurements and permits to perform express analysis of SPNP at a stage of manufacturing as well as at stage of application. The other advantage of the proposal method is simplicity. The method does not require sophisticated and costly equipment. It can be implemented with the use of much less sophisticated magnetization system when comparing to the system used in determining of magnetic nanoparticle parameters. One of the reasons of that fact is the feature of the second harmonic response dependence on the applied bias field. Since the second harmonic response is the magnetization second field derivative, all characteristic changes of the dependency take place in a narrower field region. One of the key advantages of the method when comparing with widely used method of dynamic light scattering is the possibility to determine the core of the particle when applied to the core-shell particles.

## Conclusions

The new method of the superparamagnetic nanoparticle size lognormal distribution parameter determination is proposed. Its advantage is the relative simplicity of measurement in limited field range and higher sensitivity for narrow distributions than approximation of magnetization curve. It allows obtaining a size distribution parameters of magnetic core for composite core-shell particles that is more difficult to do by means of electron microscopy [12] or dynamic light scattering [16] techniques. If this method is applied to recurring magnetic nanoparticles characterization, it does not need involving other techniques like X-ray or neutron diffraction line profile analysis [17].
